# Psychiatric Manifestations as the Primary Presentation of Frontal Meningioma

**DOI:** 10.31486/toj.22.0112

**Published:** 2023

**Authors:** Jill Lally, David Galarneau

**Affiliations:** ^1^ The University of Queensland Medical School, Ochsner Clinical School, New Orleans, LA; ^2^ Department of Psychiatry, Ochsner Clinic Foundation, New Orleans, LA

**Keywords:** *Anxiety*, *apathy*, *brain neoplasms*, *depression*, *meningioma*, *neuroimaging*

## Abstract

**Background:** Although some patients with primary brain lesions remain clinically asymptomatic, others may experience a range of symptoms, including headaches, seizures, focal neurological deficits, changes in baseline mental function, and psychiatric manifestations. Distinguishing between a primary psychiatric illness and symptoms of a primary central nervous system tumor can be especially difficult for patients with a history of mental illness. A major challenge in effectively treating patients with brain tumors is first obtaining the diagnosis.

**Case Report:** A 61-year-old female with a medical history significant for bipolar 1 disorder with psychotic features, generalized anxiety, and previous psychiatric hospitalization presented to the emergency department with worsening depressive symptoms and without focal neurologic deficits. She was initially placed on a physician's emergency certificate for grave disability, with anticipated discharge to a local inpatient psychiatric facility once she was stabilized. A frontal brain lesion, concerning for a meningioma, was found on magnetic resonance imaging and she was instead transferred to a tertiary center for urgent neurosurgical consultation. Bifrontal craniotomy with neoplasm excision was performed. The patient's postoperative course was uneventful, and continued symptom improvement was noted at the patient's 6- and 12-week postoperative visits.

**Conclusion:** This patient's clinical course exemplifies the clinical ambiguity associated with brain tumors, the challenge of obtaining a timely diagnosis with nonspecific symptoms, and the importance of neuroimaging for patients presenting with atypical cognitive symptoms. This case report contributes to the literature about the psychiatric manifestations of brain lesions, especially in patients with concurrent mental health disorders.

## INTRODUCTION

Although primary brain tumors account for only a small fraction of all malignancies, they result in an average of 16,853 deaths per year in the United States.^1^ Symptoms depend on tumor location, growth rate, and compression of adjacent structures.^2^ Not uncommonly, patients can remain clinically asymptomatic, but symptoms can include headache, seizures, focal deficits, slight changes in baseline mental function, and psychiatric manifestations. Psychiatric symptoms associated with brain tumors can manifest in many ways, including apathy, depression, mania, anxiety, hallucinations, and abulia. Keschner et al reported that 18% of patients with brain tumors (n=530) presented with a primary psychiatric complaint, while 78% of patients had at least 1 psychiatric symptom during their clinical course.^3^ Given the breadth of symptoms that may or may not arise secondary to a brain tumor, a major challenge in effectively treating patients with brain tumors is first obtaining the diagnosis. A provider may attribute these vague symptoms to another disorder, prolonging the symptom interval and delaying appropriate treatment.

Regarding psychiatric manifestations, providers may have difficulty determining if a patient's presenting complaint is primarily psychiatric in nature or attributable to an underlying disruption from neoplastic growth. Distinguishing between a primary psychiatric illness and symptoms of a primary central nervous system (CNS) tumor can be especially difficult for patients with a history of mental illness. For instance, if a patient presents with the chief complaint of apathy, the symptom may be attributed to major depressive disorder based on *Diagnostic and Statistical Manual of Mental Disorders, 5th Edition* criteria alone. Patients with a history of major depressive disorder may not receive additional diagnostic laboratory testing if their presentation is similar to previous episodes, given that patients with major depressive disorder are at an increased risk of recurrent depression, especially if they discontinue their antidepressant medication.^4^ A provider may obtain laboratory testing if the provider suspects a patient's symptoms may be associated with an alternative pathologic cause, such as thyroid dysfunction, anemia, or substance use disorder. Neuroimaging, however, is often extraneous for patients without red flag symptoms or focal neurologic deficits.

We present an unusual psychiatric presentation of a primary right frontal meningioma in an individual with a history of multiple psychiatric conditions.

## CASE REPORT

A 61-year-old female with a medical history significant for generalized anxiety, previous suicide attempt, and bipolar 1 disorder with psychotic features was brought to the emergency department (ED) by her husband for grave disability. Her husband described the patient as being progressively less active, with deteriorating memory and increased lethargy during the prior 3 months and often refusing to get out of bed for 20+ hours per day. He commented, “[she] has no will to live right now.” The patient experienced an episode of urinary incontinence the day prior to admission and remained in bed despite the incontinence. Her husband also reported a lack of appetite for the prior 3 days and refusal to eat or drink. The patient complained of a recent headache, relieved by naproxen.

Her husband reported that the patient had been seen in the ED on 2 separate occasions during the last few months, prior to this admission. On the first occasion, the husband reported that he called emergency medical services after his wife fell out of bed and refused to get up from the floor. According to the patient, she was placed on a brief psychiatric hold, which she described as “a misunderstanding,” and was eventually discharged. On that occasion, the patient was taken to an outside facility, and records could not be obtained. On the second occasion, the patient was taken to the ED for generalized weakness and nausea. She was found to be clinically stable, was prescribed 4 mg ondansetron as needed, and was discharged with a referral to her family medicine physician for a 1-week follow-up.

During this admission to the ED, the patient denied recreational substance use, which was confirmed on testing. She had had 1 inpatient psychiatric hospitalization at age 15 years and stated that she was diagnosed with dissociative identity disorder by a psychiatrist at an outside facility. She has a history of major depression, childhood trauma, and a suicide attempt at age 12 years. She was last seen by a psychiatrist 4 years prior and was lost to follow-up for unclear reasons.

Current outpatient medications prescribed by her family medicine physician were 100 mg bupropion HCl twice daily, 10 mg olanzapine nightly, 20 mg fluoxetine daily, 5 mg diazepam as needed, 145 μg linaclotide daily, 220 mg naproxen sodium as needed, and 40 mg omeprazole daily. According to the patient's husband, she had been noncompliant with these medications for approximately 5 months.

Review of systems was positive for lethargy, decreased activity, decreased appetite, and dysphoric mood. She denied physical symptoms of chest pain, shortness of breath, sore throat, blurry vision, nausea, vomiting, diarrhea, and rash. Psychiatric review of systems was negative for suicidal ideation, self-harm, and visual and auditory hallucinations.

Physical examination was positive for pallor and hypotension (blood pressure of 100/58 mm Hg). The remainder of the physical examination was insignificant. Full neurologic examination was reported to be unremarkable, with no focal neurologic deficits elicited. On psychiatric evaluation, her mood was described as dysphoric and she was described as having a flat affect and appearing “spacey and nonspontaneous” during the interview. Thought content was reported as appropriate, while thought process was reported as concrete, with some poverty of thought. Her recent and remote memory were decreased, with 3/3 recall immediately and 0/3 recall at 5 minutes. She was noted to have poor insight into her current state.

Urinalysis revealed positive nitrites and leukocytes, indicating a urinary tract infection (UTI). Comprehensive metabolic panel was significant for bicarbonate 18 mmol/L, glucose 126 mg/dL, and total bilirubin 1.2 mg/dL, likely indicating dehydration. All other laboratory tests (complete blood count, thyroid-stimulating hormone, ethanol, acetaminophen, drug screen panel, magnesium, creatine kinase, lactic acid, procalcitonin, and coronavirus disease 2019 rapid test) were unremarkable.

The patient was admitted to the hospital for further observation on the grounds of grave disability and hypotension. The patient was worked up for a recurrent episode of major depressive disorder, severe dehydration, and UTI. She was administered intravenous fluids for dehydration. A physician's emergency certificate (PEC) was issued with anticipated discharge to a local inpatient psychiatric facility once the patient was stabilized. In addition to restarting the patient's home bupropion HCl at a dose of 100 mg daily, the consult psychiatrist recommended an urgent magnetic resonance imaging (MRI) scan to evaluate for atrophic changes, stroke, or morphologic changes given the patient's rapid deterioration.

MRI of the brain identified 2 lesions concerning for meningiomas (Figure). A homogeneously enhancing extra-axial mass over the paramedial right frontal lobe measuring 4.0 × 3.9 × 4.2 cm with dural attachment was identified. Associated bilateral vasogenic edema was seen in the frontal lobes. Significant mass effect with posterior displacement of the frontal lobe was reported with an associated 2.1-cm leftward midline shift and right-sided uncal herniation. A second smaller 0.9-cm enhancing, extra-axial mass along the interhemispheric fissure was also identified, suggestive of either an aneurysm or another tumor. Brain parenchyma was otherwise normal. Following MRI results, the PEC was rescinded, an urgent neurosurgical consult was ordered, and the patient was immediately transferred to a tertiary hospital.

**Figure.**
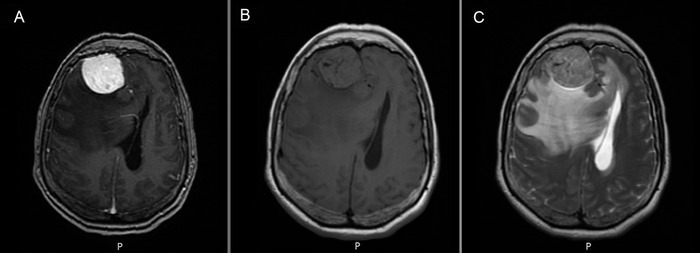
(A) T1 plus contrast, (B) T1, and (C) T2-weighted magnetic resonance axial images of the brain taken during the patient's presentation to the emergency department show a large right frontal-enhancing, well-circumscribed, extra-axial mass measuring 4.0 × 3.9 × 4.2 cm, resulting in mass effect and vasogenic edema within the right frontal lobe with an associated 2.1-cm leftward midline shift and right-sided herniation.

Upon transfer to the tertiary hospital, the patient was placed in the neurosurgical intensive care unit with neurological checks every hour. She was prescribed 500 mg levetiracetam twice daily, 4 mg dexamethasone every 6 hours, and 20 mg famotidine twice daily, and she continued this medication regimen until discharge.

Bifrontal craniotomy with neoplasm excision was performed 5 days posttransfer. Complete resection of the paramedial right frontal tumor was obtained. The smaller enhancing mass was not readily accessible.

The patient's immediate postoperative course was uneventful, and she was discharged to a rehabilitation facility 1 week postcraniotomy. Discharge medications were 100 mg bupropion HCl daily, 2 mg dexamethasone twice daily, 325 mg acetaminophen as needed, 20 mg famotidine twice daily, 5/325 mg hydrocodone/acetaminophen as needed, 100 mg lacosamide twice daily, 8.6/50 mg senna/docusate daily, 145 μg linaclotide daily, and 2 mg nicotine polacrilex as needed.

At the patient's 6-week neurosurgery follow-up, she reported improvement in her lethargy and detachment. However, she complained of ongoing headaches, forgetfulness, and nausea. Physical examination was insignificant. Reflexes were normal. Scalp incision was described as healing appropriately. Computed tomography (CT) scan was described as satisfactory with resolving edema. MRI of the brain was scheduled for 6 weeks.

Satisfactory CT and MRI scans were reported at the patient's 12-week neurosurgery follow-up, with resolving edema present. The patient noted feeling somewhat “sleepy and fatigued” but less so than prior to her surgery. She did not endorse other complaints. She was found to be neurologically intact on examination, without focal deficits. MRI of the brain showed marked regression of the right hemispheric edema with compensatory enlargement of the frontal horns of the ventricles and no clear evidence of residual tumor. The 0.9-cm enhancing parafalcine nodule was still present and may represent a second small meningioma or small aneurysm. The patient's next follow-up is scheduled for 1 year postoperatively; another MRI scan will be obtained at that follow-up.

The patient did not follow up with a psychiatrist postoperatively. Her psychiatric conditions of anxiety and bipolar 1 disorder are managed by her family medicine physician, whom she saw 6 months following her surgery.

## DISCUSSION

This case describes a patient who presented with severe apathy and depression and without focal neurologic deficits on physical examination. She reported presenting to an ED on 3 separate occasions since the onset of her symptoms and having been discharged on the first 2 occasions. Given this patient's history of bipolar 1 disorder with depressive episodes, her initial discharges, followed by the PEC and psychiatric hold, do not deviate from clinical norms. Yet her brain tumor was not identified until neuroimaging was performed during her third visit to the ED. Her clinical course exemplifies the clinical ambiguity associated with brain tumors and the challenge of obtaining a timely diagnosis with nonspecific symptoms. In fact, delays in diagnosis of primary brain tumors are a well-known barrier to surgical outcome and patient survival.^5^

Lesions within the brain can alter surrounding neurons, compress nearby structures, and disrupt neuronal activity, resulting in aberrant connectivity. Some studies have demonstrated a correlation between tumor location and clinical manifestations.^3^ Frontal and temporal region tumors have been reported to cause more psychiatric symptoms than those localized to the parietal and occipital lobes.^6^ Our patient's lesions were located on the right subfrontal lobe and the left anterior aspect of the falx. Numerous case reports and literature reviews have described an association between frontal meningiomas and depressive symptoms.^7-19^ One report described a positive association between right frontal meningiomas and rates of major depressive disorder, atypical depression, and psychosis.^20^ Peng et al analyzed 65 patients with meningiomas and categorized them based on location: frontal lobe vs nonfrontal lobe. The study found a stronger association between the medial frontal region and apathy, relative to dorsolateral frontal, ventral frontal, and nonfrontal regions.^21^ Overall, given the location and timing of our patient's symptoms—without a known change in external factors—a reasonable suggestion is that her psychiatric symptoms were a consequence of her tumor.

Nevertheless, the possibility of parallel disease manifestations cannot be excluded. The primary etiology of her psychiatric symptoms could be attributable to a concurrent psychiatric disorder and not the meningioma. The possibility of concurrent diseases is especially important to consider given the patient's history of depression and her medication noncompliance. Although continued symptom improvement was noted at the patient's 6- and 12-week postoperative visits, determining whether surgical resection or medical management was the primary antidote may be impossible. Improvement secondary to craniotomy suggests the symptoms were related to her malignancy; however, improvement secondary to bupropion therapy may indicate an organic psychiatric cause. Alternatively, her presentation may have been a combined effect.

Regardless of primary cause, neuroimaging was critical to the diagnosis. Quality evidence to recommend routine neurologic imaging in patients presenting with psychiatric symptoms is lacking.^22^ Imaging is not without inherent risks, including radiation and cost. Given the rarity of brain lesions relative to primary psychiatric conditions, these risks are likely not worth the benefit for the majority of patients. Despite the risks, however, the benefit is clear for individuals with symptoms secondary to life-threatening pathologies. The challenge lies in identifying which patients will benefit from neuroimaging. Given the aggressive nature and poor prognosis of many CNS tumors, prompt diagnosis and appropriate neurosurgical removal are key to survival.^5^ For this reason, some researchers speculate that all patients with changes in baseline mental function or a change in psychiatric symptoms should undergo neuroimaging.^22^

This case report highlights the importance of extensive diagnostic workups for patients with unusual or worsening psychiatric symptoms. Specifically, this patient's symptom interval could have been significantly prolonged if an MRI had not been ordered. Minimizing symptom interval and diagnostic delays in patients with brain tumors has been shown to increase patient survival, and thus is a priority.^5^ Given the spectrum of potential symptoms and nonspecific presenting complaints, a quick diagnosis may seem unfeasible. Additional studies are needed to assess which patients warrant neuroimaging on initial presentation. This determination is especially important for patients with relapsing or recurrent episodes of well-established disease, such as the case with our patient.

## CONCLUSION

We describe the unusual clinical presentation of a patient found to have a primary meningioma in the absence of focal neurologic deficits. Her primary symptoms subsided following surgical intervention and antidepressive medical management. This case highlights the importance of neuroimaging for individuals with acute onset or atypical psychiatric symptoms, regardless of medical history. We recommend further studies to assess the role of neuroimaging for patients presenting with worsening or altered psychiatric symptoms.

## Notes

*This article meets the Accreditation Council for Graduate Medical Education and the American Board of Medical Specialties Maintenance of Certification competencies for Patient Care and Medical Knowledge.*
